# Our experience with the use of the Interleukin-1 receptor antagonist in the treatment of the patient with CINCA/NOMID syndrome (case study - 5 years of therapy)

**DOI:** 10.1186/1546-0096-9-S1-P13

**Published:** 2011-09-14

**Authors:** V Vargova, Z Macejova

**Affiliations:** 1 1st Dep. of Pediatrics, Safarik University and Children Hospital, Kosice, Slovakia; 2 3rd Dep. of Internal Medicine, Safarik University and University Hospital, Kosice, Slovakia

## Background

CINCA/NOMID is an autoinflammatory disorder characterized by the triad: neonatal onset of cutaneous symptoms, chronic meningitis, and recurrent fever. It belongs to the group of cryopyrinopathies - it is a rare hereditary autoinflammatory syndrome caused by excessive production of interleukin-1beta.

## Objectives

To evaluate effectiveness and safety of the 5 year therapy with interleukin -1 receptor antagonist in patient with CINCA/NOMID syndrome.

## Methods

We present the case of the patient who had been monitored closely from the fifth day of her life because of the presence of neonatal exanthem, bouts of fevers, and positive inflammatory parameters. Over time the patient developed other symptoms such as pain and swelling of joints, headaches, lymphadenopathy, aseptic meningitis, and hearing impairment. Genetic analysis at the age of 24 years confirmed suspected mutation in the gene NLPR3(CAIS1) - T405P. In September 2005 the therapy with anakinra was started at the dose of 100 mg/day. Effectiveness of the therapy was evaluated on the basis of patient's subjective complaints (exathem, headaches, pain in joints, changes in well-being), objective findings and laboratory markers of inflammation.

## Results

During the first week of therapy the patient reported dramatic relief of symptoms she suffered for years (exanthem, headache, pain in joints). Gradually, in the course of 6 months of therapy, levels of inflammation markers were decreased and bouts of fever were eliminated permanently. At present the patient is monitored closely, she is free of significant subjective symptoms. Nevertherless, the loss of vision and impairment of hearing were found to be progressing. The patient tolerates without problems subcutaneous anakinra injections administered daily. Recurrent cystitis and mycotic infection of nails were treated successfully on out-of- hospital basis. Dynamics of the selected laboratory indicators (before initiation of the therapy, after 8 weeks, 6 months, and 60 months of therapy, respectively) is as follows:

ESR 70/100, 40/60, 45/52, 20/50, CRP (mg/l) 60, 7, 5, 0.44, IgG (g/l) 21.2, 19.8, 15.4, 12.5, IgA (g/l) 6.7, 5.9, 3.2, 2.5, s-creatinin (umol/l): 48, 52, 58, 65.3, u-protein: negat-negat-negat-negat.

## Conclusions

Therapy with anakinra in patient with CINCA/NOMID resulted in permanent relief of everyday clinical symptoms as well as decrease in levels of inflammation indicators (except for the ESR value which remains mildly increased). In our patient, at the age of 30, results of kidney function tests are within normal range. Our attempts to reverse progression of hearing loss were not successful (according to our opinion this is due to relatively late initiation of the therapy). We can conclude that the therapy with anakinra was effective (significantly improved the quality of life of our patient), well tolerated, and safe.

